# *Limosilactobacillus reuteri* DSM 17938 reverses gut metabolic dysfunction induced by Western diet in adult rats

**DOI:** 10.3389/fnut.2023.1236417

**Published:** 2023-10-16

**Authors:** Jumana Abuqwider, Angela Di Porzio, Valentina Barrella, Cristina Gatto, Giuseppina Sequino, Francesca De Filippis, Raffaella Crescenzo, Maria Stefania Spagnuolo, Luisa Cigliano, Gianluigi Mauriello, Susanna Iossa, Arianna Mazzoli

**Affiliations:** ^1^Department of Agricultural Sciences, University of Naples Federico II, Naples, Italy; ^2^Department of Biology, University of Naples Federico II, Naples, Italy; ^3^NBFC, National Biodiversity Future Center, Palermo, Italy; ^4^Department of Bio-Agrofood Science, Institute for the Animal Production System in the Mediterranean Environment, National Research Council Naples (CNR-ISPAAM), Naples, Italy

**Keywords:** gut, microbiota, microencapsulation, inflammation, oxidative stress

## Abstract

**Introduction:**

Microencapsulation of probiotic bacteria is an efficient and innovative new technique aimed at preserving bacterial survival in the hostile conditions of the gastrointestinal tract. However, understanding whether a microcapsule preserves the effectiveness of the bacterium contained within it is of fundamental importance.

**Methods:**

Male Wistar rats aged 90 days were fed a control diet or a Western diet for 8 weeks, with rats fed the Western diet divided into three groups: one receiving the diet only (W), the second group receiving the Western diet and free *L. reuteri* DSM 17938 (WR), and the third group receiving the Western diet and microencapsulated *L. reuteri* DSM 17938 (WRM). After 8 weeks of treatment, gut microbiota composition was evaluated, together with occludin, one of the tight junction proteins, in the ileum and the colon. Markers of inflammation were also quantified in the portal plasma, ileum, and colon, as well as markers for gut redox homeostasis.

**Results:**

The Western diet negatively influenced the intestinal microbiota, with no significant effect caused by supplementation with free and microencapsulated *L. reuteri*. However, *L. reuteri*, in both forms, effectively preserved the integrity of the intestinal barrier, thus protecting enterocytes from the development of inflammation and oxidative stress.

**Conclusion:**

From these whole data, it emerges that *L. reuteri* DSM 17938 can be an effective probiotic in preventing the unhealthy consequences of the Western diet, especially in the gut, and that microencapsulation preserves the probiotic effects, thus opening the formulation of new preparations to be able to improve gut function independent of dietary habits.

## 1. Introduction

The human environment, behaviors, and lifestyle have all dramatically changed in the last 50 years. Unhealthy dietary patterns and sedentary behavior are risk factors for major chronic diseases (1), becoming a major public health concern due to their global prevalence (2). In this context, the Western diet, in particular, is considered a major risk factor (3) due to its high content of energy, fat, and sugar. As a matter of fact, this diet model seems to exert a negative effect on the richness and function of gut microbiota, leading to a significant decrease in the number of commensal bacteria and reducing microbial diversity (4, 5). Moreover, the shift in microbiota composition can, in turn, lead to inflammation, impairment of gut function, and alteration of the immune system in this organ (4, 6).

Physical, psychological, pharmaceutical, and dietary therapies have been proposed to manage gut dysfunction (7). Among these therapies, the administration of probiotics as potential biotherapeutics has become of central interest, considering their ability, when given in sufficient amounts, to be beneficial to the host by improving gut microbial balance (8) and modulating their interaction with intestinal cells, with positive effects on the health of the whole organism (9). However, since the physiological effects attributed to probiotics are highly strain-specific, their selection may be critical for their functional efficacy (10). Therefore, each strain may work through different mechanisms of action and have a specific genotype/phenotype accordingly, and these mechanisms are not deeply and entirely understood yet (11).

A major selection criterion for a probiotic is its capacity to pass through the human gastrointestinal tract as undamaged as possible to reach the gut and exert its activity (12, 13). Indeed, the insufficient viability and survival of probiotics remain pivotal in supplements and foods (13). Constructing a probiotic release system is extremely complex due to the diverse range of conditions in the human digestive tract. However, this diversity also presents an opportunity to develop a customized system that specifically targets the intended gut site (14).

The bacteria's survivability can be improved by the microencapsulation technique. The microencapsulation matrix is designed to resist the gastrointestinal acid condition, and the preparation routine must be delicate to not harm the cells within it. Furthermore, the polymer utilized must be non-cytotoxic and non-antimicrobial to ensure that neither the host nor the bacteria are damaged (15). This technique has been successfully applied in other oral delivery applications, proving how the specialized ultrathin semipermeable polymer membranes that constitute the microcapsule can protect live bacterial cells (16). *Limosilactobacillus reuteri* DSM 17938 (*L. reuteri*), known as *Lactobacillus reuteri* before the changes in the taxonomy of the *Lactobacillus* genus, deeply revised in 2020 (17), is a probiotic strain that can colonize several human body sites, such as the gastrointestinal tract, the skin, breast milk, and the urinary tract. Several studies reported in the literature showed that *L. reuteri* administration can be beneficial to human health and disease (18). In particular, it is a probiotic well recognized for its useful effects in pediatric gastrointestinal diseases, especially in children (19, 20) and in cases of constipation in adults (21), but, to date, only two studies have investigated its effect in the context of obesity and metabolic syndrome (22, 23).

The overall aim of this study has been to evaluate the effects of free or microencapsulated probiotic *Limosilactobacillus reuteri* DSM 17938 on the intestinal microbiota and the gut metabolic alteration induced by a Western diet that combines the consumption of a high saturated fat diet with high fructose intake, perfectly simulating the current dietary habits in Western countries. In particular, we focused on the integrity of the intestinal barrier, gut inflammation, oxidative stress, and enzymes playing a critical role in redox homeostasis and lipopolysaccharide (LPS) detoxification.

## 2. Materials and methods

### 2.1. Cultivation and microencapsulation of *Limosilactobacillus reuteri* DSM 17938

*Limosilactobacillus reuteri* DSM 17938 was kindly provided by BioGaia (Noos S.r.l.; BioGaia AB, Stockholm, Sweden). It was cultured in MRS broth (OXOID Ltd., Basingstoke, Hampshire, England) at 37°C, checked for purity, and kept on MRS Agar (OXOID Ltd., Basingstoke, Hampshire, England). The free and microencapsulated cells were cultured in aerobic conditions and counted routinely on MRS agar at 37°C for 48 h. The encapsulator B-395 Pro was equipped with a 120-mm nozzle and a syringe pump (BÜCHI Labortechnik, Flawil, Switzerland) to microencapsulate bacterial cells. The number of microencapsulated cells was determined according to De Prisco et al. (24), and the entrapment efficiency was calculated using the formula:


$$\begin{array}{l} N=Nd/Ni, \end{array}$$

where N is the average number of CFU per microcapsule, Nd is the cell count (CFU/ml) of disrupted microcapsules, and Ni is the cell count of integer microcapsules. Each colony is derived from a single integer microcapsule; therefore, Ni's measure unit is microcapsules/ml (25).

### 2.2. Animals and treatments

All animal experiments were authorized by the Italian Health Ministry (137/2022-PR) and approved by the “Comitato Etico-Scientifico per la Sperimentazione Animale” of the University of Naples “Federico II.” The procedures used in this work adhere to the animal ethics principles and regulations of the Italian Health Ministry. The authors ensured that all steps were taken to minimize the pain and suffering of the animals.

Male Wistar rats (Charles River, Calco, Lecco, Italy) aged 90 days were caged in a temperature-controlled room (23 ± 1°C) with a 12-h light/dark cycle (06.30–18.30 h).

Rats were divided into four groups (each composed of 8 rats) and treated for 8 weeks with a control diet (C group) or with a high-fat, high-fructose diet (W, WR, and WRM groups). In addition, WR rats daily received 0.1 mL of a 10% sucrose solution containing 10^8^ CFU of *L. reuteri*, WRM rats daily received 0.1 mL of a 10% sucrose solution containing 10^8^ CFU of microencapsulated *L. reuteri*, and C and W rats received the same amount of sucrose solution without probiotics. Sucrose solution with or without probiotics was presented by an operator every day at the same hour through a needless syringe and voluntarily consumed by rats. We also calculated that the quantity of alginate per dose is 6 mg. The two diets are shown in Supplementary Table S1. During the treatment, body weight and food intake were monitored, and the feces were collected daily. The results are reported in Supplementary Figure S1. After 8 weeks on the diet, the rats were euthanized, and portal blood, colon, and ileum were taken and frozen at −80°C for further analysis.

### 2.3. DNA extraction, high-throughput sequencing, and bioinformatic analysis

Fresh fecal samples of 32 rats (8 for each of the four groups) were collected after 8 weeks of treatment. The DNeasy PowerSoil Pro Kit (Qiagen, Hilden, Germany) was used to extract total DNA according to the manufacturer's instructions and quantified using the NanoDrop spectrophotometer. Bacterial diversity was determined by high-throughput sequencing (HTS) of the amplicons from the V4-V3 region of the 16S rRNA gene (~460 bp). PCR and bioinformatic analysis were carried out as previously reported (26–28).

### 2.4. Markers of inflammation

Portal plasma samples were obtained from portal blood collected in tubes containing EDTA at the time of sacrifice. These samples were then centrifuged for 15 min at 1400 x g and stored at −20°C.

LPS in portal plasma was measured using a protocol based on a Limulus amoebocyte lysate (LAL) extract (ThermoFisher Scientific, Rockford, IL, USA) in accordance with the manufacturer's instructions.

Portal plasma concentrations of tumor necrosis factor-alpha (TNF-α), interleukin-6 (IL-6), and interleukin-10 (IL-10) were assessed using an enzyme-linked immunosorbent assay (R&D Systems, Minneapolis, MN, USA), specific for rats, which was in accordance with the kit instructions.

Intestinal alkaline phosphatase (IAP) activity was measured in the ileum and the colon. The ileum and colon tissues for the analysis were prepared according to Kaliannan et al. (29), and IAP activity was determined following the reported protocol (29), with the addition of the selective IAP inhibitor phenylalanine (10 mM) to subtract the result with phenylalanine from the result without phenylalanine (30). The specific activity of the enzyme is expressed as picomoles of pNPP hydrolyzed/min/μg of protein. A protein assay reagent from Fisher Scientific determined the protein concentration in each sample.

### 2.5. Oxidative stress parameters in the ileum and colon

To measure oxidative stress markers, homogenates from the colon and ileum were made in a 50 mM phosphate buffer with a pH of 7.0 (1:50 w/v).

Nitro-tyrosine (N-Tyr) levels were determined by ELISA, as described previously (31, 32).

According to Fernandes et al. (33), the thiobarbituric acid assay has been used to measure lipid peroxidation.

Superoxide dismutase (SOD) activity was assessed by following the decrease in the reduction rate of cytochrome c by superoxide radicals in a buffer with 20 mM cytochrome c, 0.1 mM xanthine, 0.01 units of xanthine oxidase, 50 mM KH_2_PO_4_, pH 7.8, as reported in a previous study (34).

Catalase activity was determined in a 50-mM phosphate buffer, pH 7.0, with 0.25% Triton X-100 and 10 mM H_2_O_2_ by following the decay of H_2_O_2_ at 240 nm, as reported in a previous study (35).

Glutathione reductase (GR) activity was measured following the decrease of NADPH absorbance at 340 nm, as described in a previous study (36).

NADPH oxidase activity was assayed by monitoring the change in absorbance at 340 nM, as previously reported (37).

### 2.6. Immunofluorescence analysis

Sections of the colon and ileum from all the groups were paraffin-embedded and stained with an occludin-specific monoclonal antibody (Invitrogen, Carlsbad, CA, USA) as a marker of gut barrier integrity. Subsequently, the slides were stained with DAPI (Sigma Aldrich, Saint Louis, MO, USA). The images were captured and visualized using a Zeiss Confocal Microscope LSM 700 at 40× magnification, and three random fields/section per rat were analyzed using ImageJ software (National Institutes of Health, Bethesda, MD, USA).

### 2.7. Western blot quantifications of TLR4 and p-NFkB in the ileum and colon

A Western Blot analysis was carried out on protein extracts from the colon and ileum to detect toll-like receptor-4 (TLR4) and phospho-nuclear factor kB (p-NFkB) signals as markers of inflammation, as previously described (38). A list of antibodies with their source and catalog number is presented in Supplementary Table S2.

### 2.8. Statistical analysis

To evaluate the presence of significant differences in the abundance of taxa between the four groups of rats, the pairwise Wilcoxon-Mann–Whitney test (“pairwise.Wilcox.test” function in package “base” was used). A *p* < 0.05 was considered statistically significant.

The animal physiological data were expressed as mean values ± SEM. GraphPad Prism 6 (GraphPad Software, San Diego, CA, USA) was utilized to check the normal distribution of the raw data and to perform a one-way ANOVA. In all analyses, a *p* < 0.05 (probability < 5%) was considered statistically significant.

## 3. Results

### 3.1. EE and cell survival

We calculated an EE of 97% and an average CFU/microcapsule of 100. The results of cell counting showed a cell load of microcapsules of 2.0 × 10^8^ CFU/ml, and this value remained constant during the storage of the microcapsules in Ringer at 4°C for more than 14 days. However, we replaced the microcapsules weekly with a fresh preparation to ensure a dose of 10^8^ CFU of *L. reuteri*.

### 3.2. Intestinal microbiota composition

**Figure 1:**
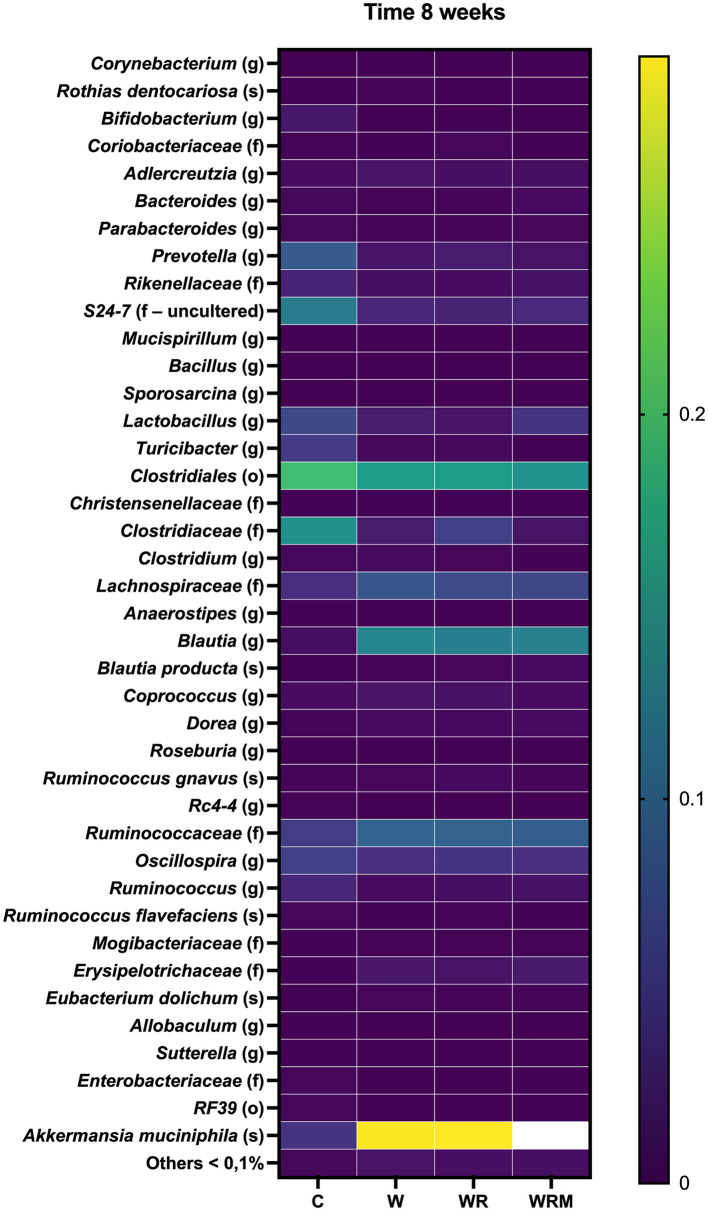
Heatmap showing average abundance (%) of the most abundant taxa (>0.1% abundance) in the gut microbiota of C, W, WR, and WRM rats. The values are the means of eight different rats.

To profile the effects of diet and *L. reuteri* supplementation on microbiota composition, we performed 16S rRNA gene sequencing of rat fecal samples collected at 8 weeks. The Western diet dramatically changed gut microbiota composition. The heat map in Figure 1 shows the average abundance of the most abundant taxa in C, W, WR, and WRM rats. Prevotella and Clostridiales levels decreased in western diet-fed rats (W), as well as in the rats fed the Western diet and treated with free (WR) and microencapsulated *L. reuteri* (WRM) groups compared to control rats (C) (*P* < 0.05), while *Blautia, Ruminococcaceae, Lachnospiraceae*, and *Akkermansia muciniphila* increased in all the groups consuming the Western diet compared to the C groups (*P* < 0.05). However, we did not observe a shift in the overall microbiota composition due to *L. reuteri* treatment. It is important to underline that we exclude any effect of the microencapsulating agent that is well known to be undigestible and not absorbable from the human intestine (39). Furthermore, it was previously ascertained that the scarce effect of alginate on mice was at doses much higher than those we used (40), and the alginate capsules proved to be highly biocompatible not only for short periods (i.e., 1 month) but also in the long term, as evidenced by the absence of any significant biological response up to 2 years after implantation in rats (41).

### 3.3. Tight junction protein content and portal plasma inflammation

**Figure 2:**
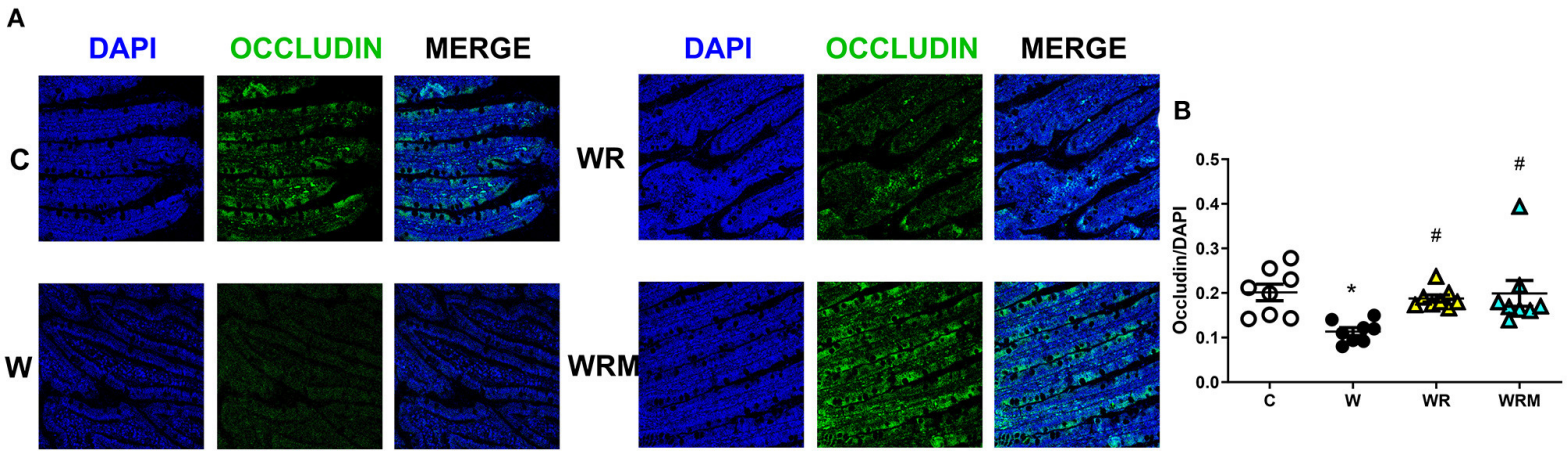
Immunofluorescence quantification of occludin (magnification 40x, scalebar = 50 μm) in the ileum **(B)** with representative images **(A)**. Values are expressed as the means ± SEM of eight rats. *P < 0.05, compared to C; ^#^*P* < 0.05, compared to W (one-way ANOVA followed by a Bonferroni post-test).

**Figure 3:**
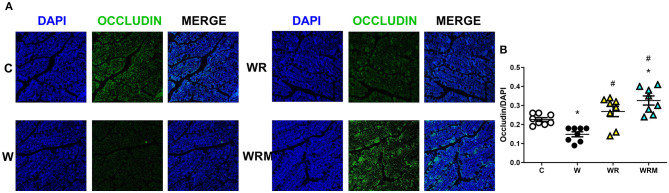
Immunofluorescence quantification of occludin (magnification 40x, scalebar = 50 μm) in colon **(B)** with representative images **(A)**. Values are expressed as the means ± SEM of eight rats. *P < 0.05, compared to C; ^#^*P* < 0.05, compared to W (one-way ANOVA followed by a Bonferroni post-test).

Tight junction occludin protein content was significantly lower in the ileum (Figure 2) and the colon (Figure 3) of W rats. In contrast, there was a significantly higher occludin content in WR and WRM rats (Figures 2, 3) in the ileum and colon compared to W rats, with even higher levels in WRM rats compared to C.

**Figure 4:**
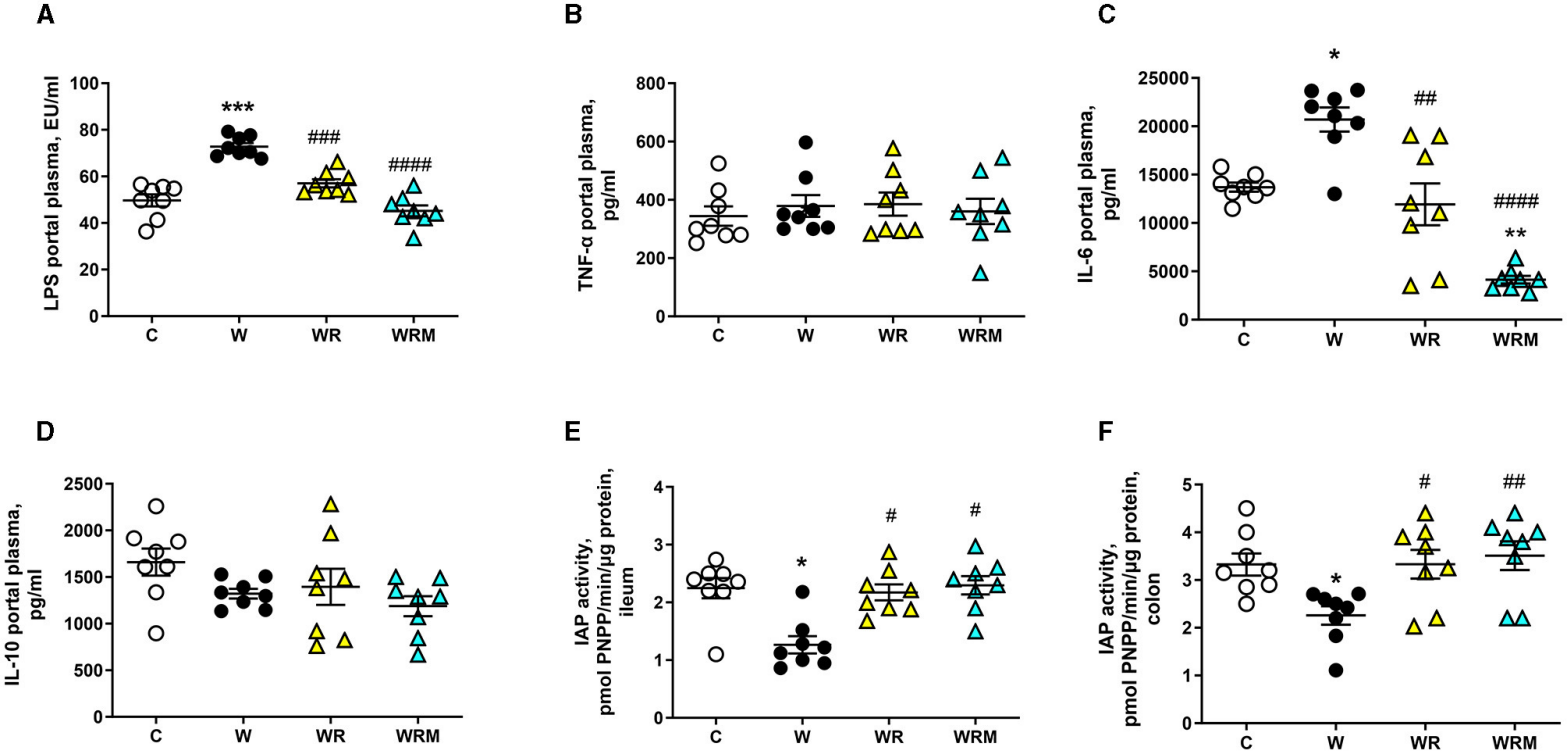
Portal plasma levels of lipopolysaccharides (LPS) **(A)**, TNF-α **(B)**, IL-6 **(C)**, and IL-10 **(D)** and intestinal alkaline phosphatase (IAP) **(E, F)**. Values are expressed as the means ± SEM of eight rats. ^*^P < 0.05, ^**^P < 0.01, ^***^P < 0.001 compared to **(C)**; ^#^*P* < 0.05, ^##^*P* < 0.01, ^###^*P* < 0.001, ^####^*P* < 0.0001 compared to W (one-way ANOVA followed by Bonferroni post-test).

The altered integrity of the gut barrier induces the development of the so-called “leaky gut” (42), determining the uncontrolled passage of substances, such as LPS, in the portal plasma. We found increased levels of LPS in the portal plasma of W rats (Figure 4A), associated with the increase of proinflammatory IL-6 (Figure 4C). These parameters were comparable to control rats in WR rats, while in WRM rats, they were even significantly improved compared to C (Figures 4A, C). No changes were found in the levels of TNF-α, a proinflammatory cytokine, and IL-10, an anti-inflammatory one, in the four groups of rats (Figures 4B, D).

IAP activity has been evaluated as an anti-inflammatory marker, considering its ability to detoxify LPS and other bacterial toxins. In agreement with the results obtained on portal LPS levels, IAP activity was found to be significantly lower in the ileum and colon of W rats compared to the control group, while it was unaltered in the above tissues of WR and WRM rats (Figures 4E, F).

### 3.4. Modulation of the inflammatory response in the ileum and colon

**Figure 5:**
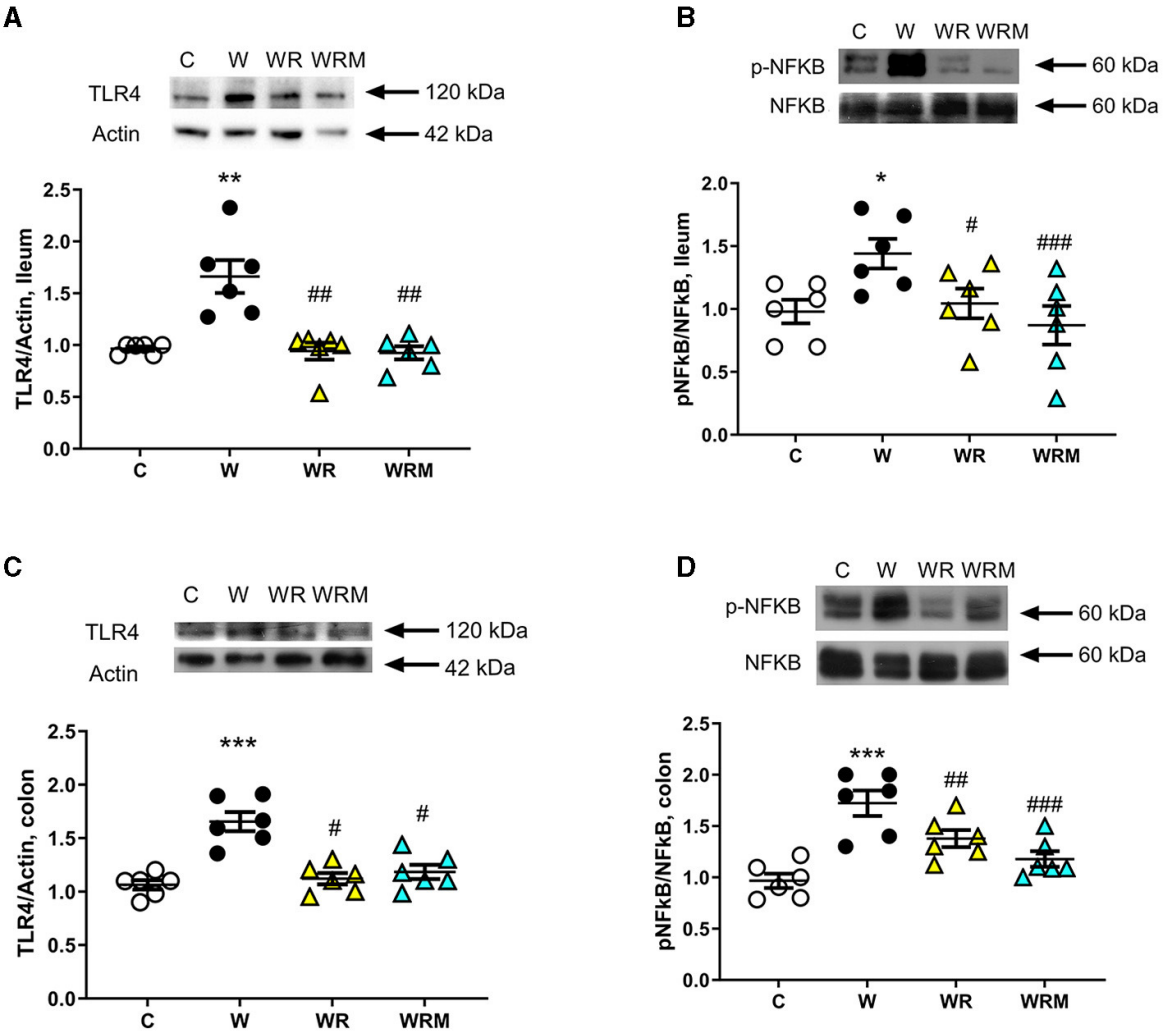
The protein content of toll-like receptor 4 (TLR4) and the degree of phosphorylation of nuclear factor kB (NFkB) (with representative blots, normalized to controls) in the ileum **(A, B)** and colon **(C, D)**. Values are expressed as the means ± SEM of six rats. *P < 0.05, **P < 0.01, ***P < 0.001, compared to **(C)**; ^#^*P* < 0.05, ^##^*P* < 0.01, ^###^*P* < 0.001 compared to W (one-way ANOVA followed by Bonferroni post-test).

TLR4 protein content in the ileum and colon was assessed as a marker of inflammation and the degree of activation of the transcription factor NFκB. In the ileum and colon, the Western diet caused an increase in TLR4 levels (Figure 5A), together with a higher degree of phosphorylation and, hence, the activation of NFκB (Figure 5B). All the above increases were prevented by the administration of *L. reuteri*, both free and microencapsulated.

### 3.5. Ileum and colon oxidative stress

The gut inflammatory status is strictly related to oxidative stress in the enterocyte (43). Therefore, we evaluated, in both the ileum and colon, the oxidative stress induced by the Western diet by measuring lipid peroxidation and N-Tyr as markers of damage to lipids and proteins, respectively, and NADPH oxidase activity as a source of reactive oxygen species (ROS) and the activity of GSR, catalase, SOD, as antioxidant enzymes.

**Figure 6:**
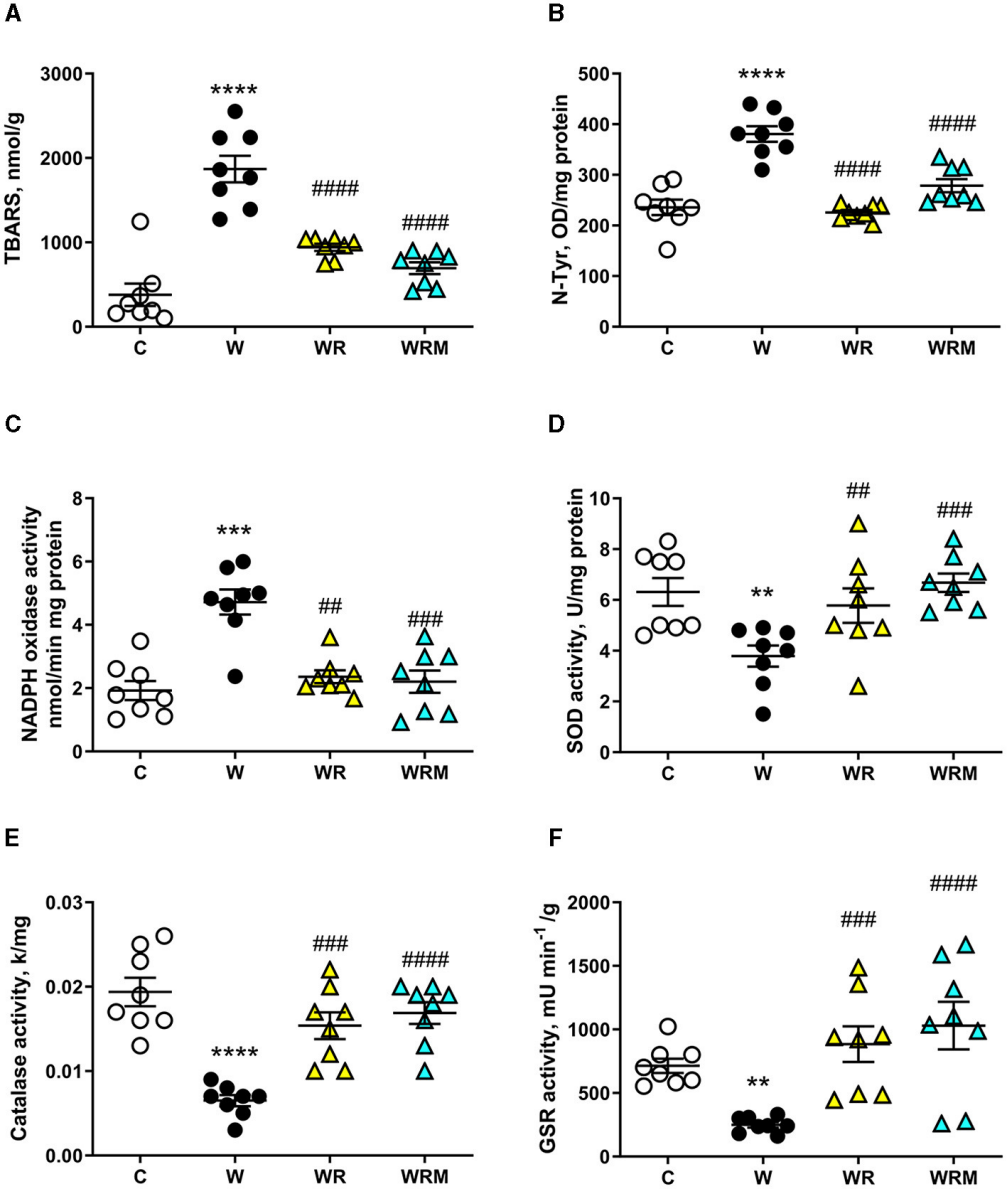
Thiobarbituric acid reactive substances (TBARS) **(A)** and N-Tyr **(B)** levels, NADPH oxidase **(C)**, superoxide dismutase (SOD) activity **(D)**, catalase **(E)**, and glutathione reductase (GSR) **(F)** activities in the ileum. Values are expressed as the means ± SEM of eight rats. **P < 0.01, ***P < 0.001, ****P < 0.0001 compared to **(C)**, ^##^*P* < 0.01, ^###^*P* < 0.001, ^####^*P* < 0.0001 compared to W (one-way ANOVA, followed by Bonferroni's post-test).

In particular, we observed significantly higher levels of TBARS and N-Tyr in the ileal cells of rats fed the Western diet (Figures 6A, B), together with an increase in NADPH oxidase activity (Figure 6C) and a decrease in GSR, catalase, and SOD activities (Figures 6D–F). Interestingly, the administration of both free and microencapsulated *L. reuteri* prevented the onset of oxidative damage since TBARS, N-Tyr levels, and NADPH oxidase activity were comparable to the control rats (Figures 6A–C). In agreement with these results, the analysis of SOD, catalase, and GSR (Figures 6D–F) activities in the ileum showed an increase in WR and WRM rats compared to W rats, confirming the beneficial effect of *L. reuteri*, both free and microencapsulated, in protecting this tissue from oxidative stress.

**Figure 7:**
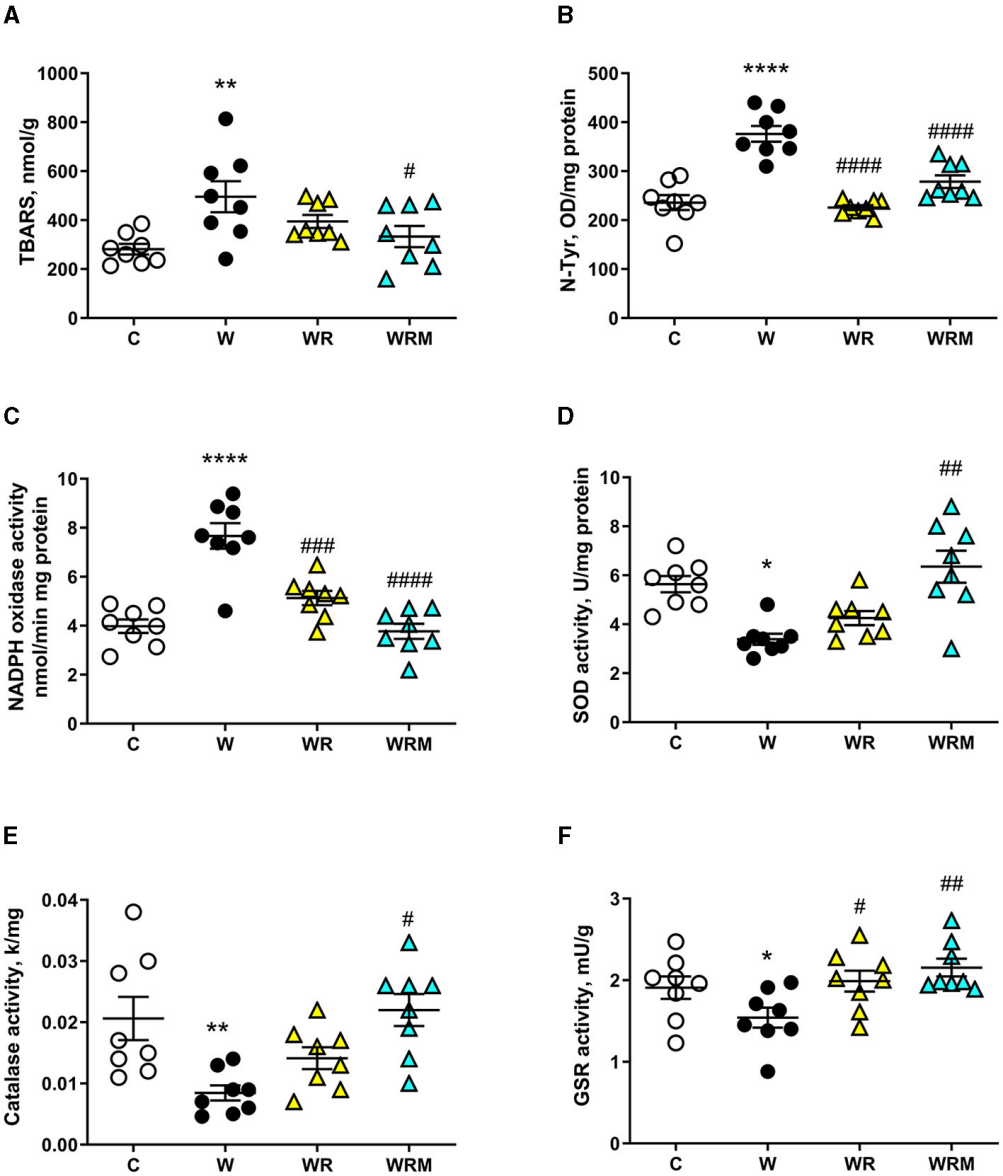
Thiobarbituric acid reactive substances (TBARS) **(A)** and N-Tyr **(B)** levels, NADPH oxidase **(C)**, superoxide dismutase (SOD) activity **(D)**, catalase **(E)**, and glutathione reductase (GSR) **(F)** activities in the colon. Values are expressed as the means ± SEM of eight rats. *P < 0.05, **P < 0.01, ****P < 0.0001 compared to **(C)**; ^#^*P* < 0.05, ^##^*P* < 0.01, ^###^*P* < 0.001, ^####^*P* < 0.0001 compared to W (one-way ANOVA, followed by Bonferroni's post-test).

A slightly different pattern was found in the colon. Indeed, while N-Tyr levels and the activity of GSR and NADPH oxidase were positively influenced by treatment with both free and microencapsulated *L. reuteri* (Figures 7B, C, F), the activities of catalase and SOD remained significantly lower (Figures 7D, E) and the levels of TBARS remained significantly increased (Figure 7A) in WR rats. Therefore, in the colon, it seems that the free bacterium is less efficient than in the ileum, while it retains its efficacy in the microencapsulated form even in this intestinal area.

## 4. Discussion

In this study, we examined the effect of *Limosilactobacillus reuteri* DSM 17938 on contrasting Western diet-induced gut metabolic disturbances and clarified whether its microencapsulation would maintain unaltered or even enhance its beneficial effect.

The Western diet is widely recognized to cause gut dysbiosis, inflammation, and metabolic endotoxemia. In agreement with this, we highlighted a strong effect of the Western diet on gut microbiota composition, including a decrease in *Prevotella*, which is typically associated with high-fiber diets (44), and an increase in *Blautia, Ruminococcaceae, Lachnospiraceae*, and *Akkermansia muciniphila*, as previously found by other authors (45). Moreover, our results proved that supplementation with *L. reuteri* DSM 17938 cannot control the consequences of the Western diet on gut microbiota deviation. We found no significant differences in any of the *taxa* (*P* > 0.05) among the W, WR, and WRM groups, which is in agreement with previous studies (23, 46). Although we analyzed fecal rather than cecal microbiota, it has been shown that the fecal microbiome represents a good non-invasive proxy of the cecal microbiome, making it suitable for detecting biologically relevant patterns (47).

Western diet-induced dysbiosis is associated with many abnormalities. One of the most relevant is the disruption of the gut barrier, whose main function is to provide a physical barrier to counteract the possible bacterial incursion from the lumen of the gut (48). Moreover, several studies have shown that high-fat diet administration can alter intestinal permeability and induce endotoxemia (49, 50). We also previously demonstrated that even 3 weeks (short term) of a high fructose diet in young rats can lead to gut barrier dysfunction (38). In this study, it was demonstrated that the consumption of a Western diet over a period of 8 weeks resulted in both gut dysbiosis and a change in the permeability of the gut barrier's mechanical line of defense, as indicated by a reduction in ileum and colon occludin levels. Occludin is a crucial functional element of tight junctions, which highly regulate the movement of substances through the paracellular space and prevent uncontrolled leakage (51).

As a consequence, we found high portal plasma levels of bacterial LPS, a powerful player in the activation of the inflammatory response (52), in rats fed with the Western diet. This is congruent with some previous studies showing an increase in plasma LPS upon consumption of high-fat (53) and high-fructose diets (50). Moreover, LPS, in turn, can further impair gut permeability through the downregulation of occludin itself and induce the establishment of the “leaky gut” (54). What is interesting to notice here is that the administration of *L. reuteri* prevented the disruption of the gut barrier in WR rats and even improved occludin expression on the intestinal barrier in the colon of WRM rats. This result shows that the free *L. reuteri* supplementation is efficient in avoiding the Western diet's negative effect on intestinal barrier integrity, while the microencapsulated form of *L. reuteri* exhibits a stronger protective effect, even increasing the integrity of the intestinal barrier, compared to C rats. These effects on the gut barrier exert an effect on the passage of LPS in WR rats, whose portal levels are comparable to those of C rats and are lower in WRM rats compared to controls. The results obtained with the free form of *L. reuteri* are in agreement with studies demonstrating that treatment with different strains of *L. reuteri* leads to a higher expression of tight junction proteins in intestinal epithelial cells (55–57), consequently inhibiting the passage of LPS through the gut barrier.

Although microencapsulation is not a novel technique, vibrating technology has been introduced in the last few years. Compared to others, the main advantages of this technology are that the process is performed at room temperature, the resulting microcapsules are very homogenous in size, shape, and number of entrapped bacterial cells, and the efficiency of encapsulation is very high. In addition, the viability of the culture is not affected by the microencapsulation process, and all the microcapsules have the same behavior in terms of bacterial activity. A previous study (24) demonstrated the stability of the carrier and the encapsulated probiotic strain during the storage of microcapsules.

Although it is acknowledged that a diet rich in fat causes metabolic endotoxemia in humans and animals (53, 58), the underlying molecular mechanisms are not fully understood yet. In this context, a role has been proposed for the enzyme IAP, which is an enzyme produced exclusively in the intestine that belongs to the alkaline phosphatase family (59). IAP can prevent the recognition of LPS from its receptor TLR4, removing a phosphate group from the LPS lipid A moiety, thus detoxifying it (60). In addition, IAP can upregulate the expression of the tight junction proteins, preserving intestinal barrier integrity and function (61). Moreover, in intestinal inflammatory conditions such as IBD (inflammatory bowel disease) and T2-DM (type 2 diabetes mellitus), the endogenous IAP levels have been shown to be extremely low (62, 63). Therefore, we investigated whether the increased LPS portal levels elicited by the Western diet were correlated with changes in IAP activity. Our present results support the involvement of IAP activity since W rats exhibited lower levels of IAP activity both in the ileum and the colon, while the administration of free or microencapsulated *L. reuteri* completely reversed the above decrease.

LPS in portal plasma is recognized by the TLR4 receptor and activates a signal cascade that involves the NFkB pathway (64). Concurrently, we found an increase in the ileal and colonic TLR4, whose expression on intestinal epithelial cells has shown a proinflammatory role, recognizing specifically the LPS (65) and NFkB involved in the intestinal dysfunctional states (66). This, in turn, implies a higher gene expression of TNF-α (67) and IL-6 (68) and a consequent inflammatory status. Indeed, an increase in the inflammatory cytokine IL-6 was detected in the portal plasma of W rats compared to controls, but quite surprisingly, no differences were found in TNF-α levels among C, W, WR, and WRM rats. This result can be explained by considering that increased IL-6 levels in humans have been shown to inhibit endotoxin-induced TNF-α production (69). Moreover, attenuated TNF-α production has been shown after IL-6 treatment in plasmacytoid dendritic cells in rheumatoid arthritis (70).

Moreover, in agreement with us, unchanged TNF-α levels were found by Bedoui and colleagues in male rats fed for 10 weeks with a high-fat diet (71), and by Semeraro and colleagues in female rats fed for 10 months with a high-fat diet (72). These changes suggest additional damage to intestinal cell function and structure. It is known that IL-6 and NFkB have a key role in the regulation of tight junction integrity (73, 74). Moreover, in the rats that were fed the Western diet with the free probiotic *L. reuteri* administration, IL-6 levels, TLR4, and p-NFkB were comparable to the levels found in the C rats, confirming the hypothesis that the supplementation of a probiotic in parallel with the Western diet protects against the development of inflammation. Again, the microencapsulated *L. reuteri* exhibits a more powerful anti-inflammatory activity, with IL-6 levels lower than those found in C rats.

Oxidative stress development is often associated with intestinal inflammation (75), and several studies show a close interaction between oxidative stress and gut microbiome alterations (76). In agreement, we demonstrated that the Western diet induces oxidative damage to both proteins and lipids in the ileum and colon due to an imbalance between ROS production and the functioning of the antioxidant defense system. Indeed, we found an increase in the ROS-producing NADPH oxidase activity coupled with a decrease in the antioxidant enzymes SOD, catalase, and GSR in Western diet-fed rats compared to the control rats both in the ileum and the colon. In the ileum, we also demonstrated the efficiency of *L. reuteri* in preventing oxidative stress, acting on the inhibition of ROS production, and stimulating antioxidant defense, with only a marginal effect on the microcapsule. However, in the colon, we evidenced a strong improvement of the microcapsule in the action of the probiotic bacterium. Although we found a decrease in ROS production in WR rats, the lack of effect on catalase and SOD activities resulted in increased TBARS levels, while in WRM rats, both ROS production and antioxidant enzymes were comparable to those found in control rats. Therefore, we can speculate that the microcapsule enhances the resistance of probiotics to the acid environment in the stomach, which is severely fatal to the majority of bacteria and the bile acids and digestive enzymes in the small intestine, thus increasing the survival rate and viability and ensuring that a higher number of viable probiotics arrive in the colon, thus allowing greater effectiveness of the probiotic bacterium in this district. Previous studies performed with microencapsulated *Lactobacillus salivarious* Li01 and *Pediococcus pentosaceus* Li05 showed an enhancement of storage viability and delivery to gut microbiota (77, 78). In fact, in conjunction with its well-established role as a mere physical barrier to harsh gut conditions, microencapsulation was recently found to enhance the stress resistance of bacterial cells through the mediation of quorum-sensing activity (79). Moreover, considering that the colon has the largest bacterial density (10^11^ to 10^12^ CFU/ml), probiotics will find a high colonization resistance from commensal bacteria (80). Hence, the protection provided by the microcapsule is even more important in guaranteeing the release of a greater number of bacteria that can colonize the colon.

**Figure 8:**
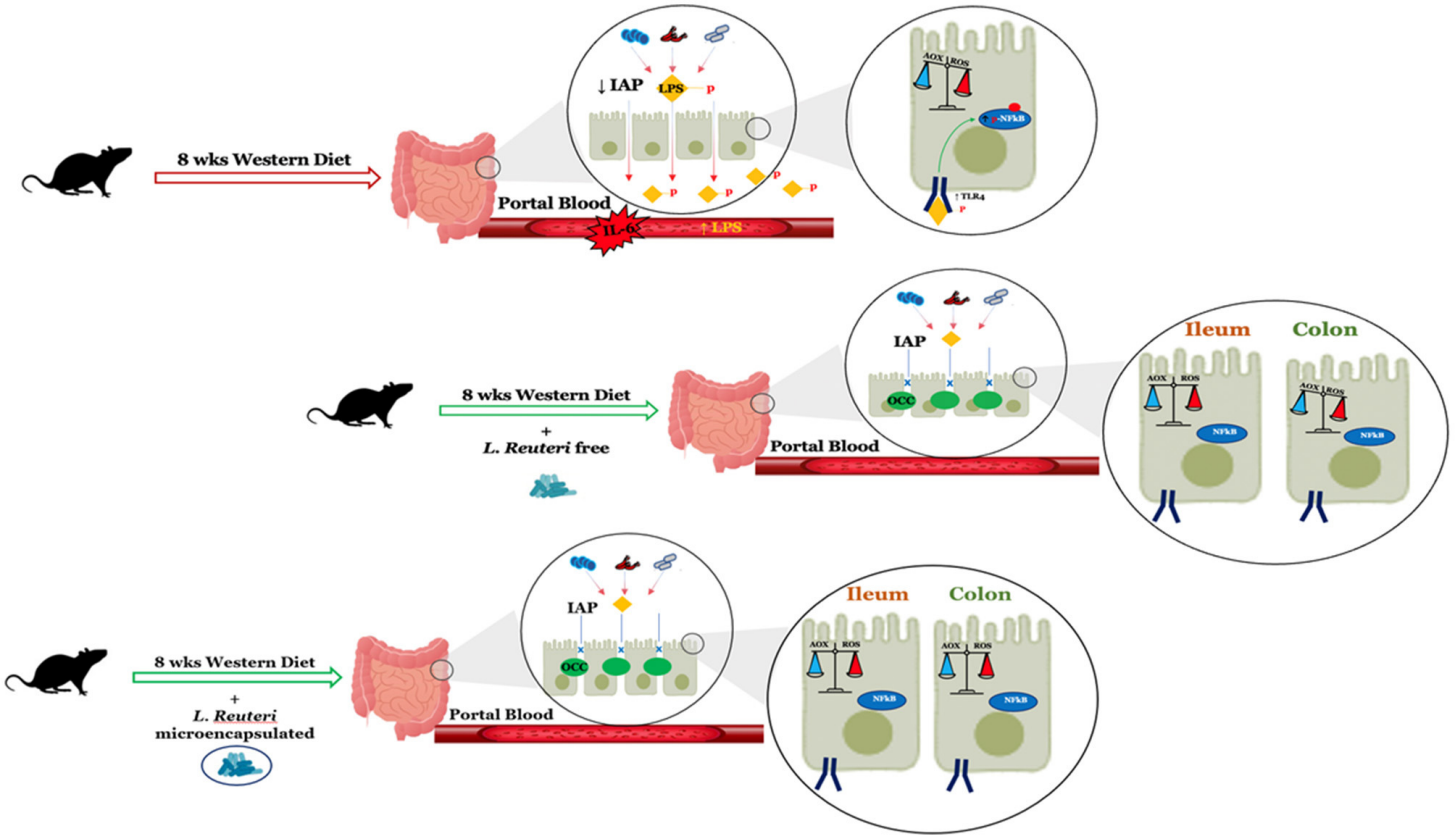
Summary of the changes in intestinal function after 8 weeks of a Western diet with or without *L. reuteri* free or microencapsulated supplementation in adult rats. LPS, lipopolysaccharide; IAP, intestinal alkaline phosphatase; OCC, occludin.

The results obtained in this study demonstrate, for the first time, the efficiency of *Limosilactobacillus reuteri* DSM 17938 treatment in improving the metabolic alterations induced in the gut by the Western diet. Indeed, the fact that *L. reuteri* safeguards intestinal permeability plays an important role in reducing low-grade inflammation induced by elevated lipopolysaccharide levels and oxidative stress in enterocytes. Moreover, our data also demonstrate how microencapsulation not only does not alter the effectiveness of the bacterium but even improves it, especially in the colon (Figure 8). From these whole data, it emerges that *L. reuteri* DSM 17938 can be an effective probiotic in preventing the unhealthy consequences of the Western diet, especially in the gut, and that microencapsulation preserves the probiotic effects, thus opening up the formulation of new preparations that can improve gut function independent of dietary habits.

## Data availability statement

The original contributions presented in the study are included in the article/Supplementary material, further inquiries can be directed to the corresponding author.

## Author contributions

GM, SI, and AM: conceptualization and supervision. JA, SI, and AM: data curation. SI, AM, and GM: funding acquisition. JA, AD, VB, CG, GS, FD, RC, and MS: investigation. JA, SI, GM, and AM: writing—original draft. JA, AD, VB, CG, GS, FD, RC, MS, LC, GM, SI, and AM: writing—review and editing. All authors contributed to the article and approved the submitted version.
