# Supplementary material for: Limosilactobacillus reuteri DSM 17938 reverses gut metabolic dysfunction induced by Western diet in adult rats
Source: Front Nutr. 2023 Oct 16;10:1236417. doi: 10.3389/fnut.2023.1236417 (PMC10613642; doi:10.3389/fnut.2023.1236417)
Supplement: Supplementary file 1 [file Data_Sheet_1.PDF]

Supplementary Material

Microencapsulated *Limosilactobacillus reuteri* DSM 17938 reverses gut metabolic dysfunction induced by Western diet in adult rats

Jumana Abuqwider[1], Angela Di Porzio[2], Valentina Barrella[2, 4], Cristina Gatto[2], Giuseppina Sequino[1], Francesca De Filippis[1], Raffaella Crescenzo[2], Maria Stefania Spagnuolo[3], Luisa Cigliano[2], Gianluigi Mauriello[1], Susanna Iossa[2, 4], Arianna Mazzoli[2*]

*** Correspondence:** Arianna Mazzoli arianna.mazzoli@unina.it

1 Supplementary Table

Table S1. Diet composition

Component	Composition g/1000 g	
	Low Fat	Western diet
Standard Chow[a]a	395.3	231.5
Sunflower oil	19.3	19.3
Casein	59.7	133.3
Water	175.7	175.4
AIN-93 Mineral mix	11.4	11.4
AIN-93 Vitamin mix	3.2	3.2
Choline	0.7	0.7

**Methionine**	0.9	0.9
**Cornstarch**	333.8	0
**Butter**	0	129.8
**Fructose**	0	294.6
**Energy content and composition**		
**ME content, kJ/g[b]**	11.2	14.9
**Lipids, J/100 J**	10.5	39.3
**Proteins, J/100 J**	19.9	19.8
**Complex carbohydrates, J/100 J**	63.9	7.5
**Simple sugars, J/100 J**	5.7	33.4

[a]4RF21, Mucedola, Italy; [b]Estimated by computation using values (kJ/g) for energy content as follows: Protein 16.736, lipid 37.656, and carbohydrate 16.736. ME = metabolizable energy; AIN= American Institute of Nutrition.

Table S2: Source of antibodies.

ANTIBODIES	*SOURCE*	*CATALOG NUMBER*
ABII HRP MOUSE	PROMEGA	W4011
ABII HRP RABBIT	PROMEGA	W4021
NFKB	SANTACRUZ	Sc-8008
ACTIN	SIGMA-ALDRICH	A2066
p-NFKB p65 (27.Ser536)	SANTACRUZ	Sc-136548
TRL4	SIGMA-ALDRICH	PRS3141

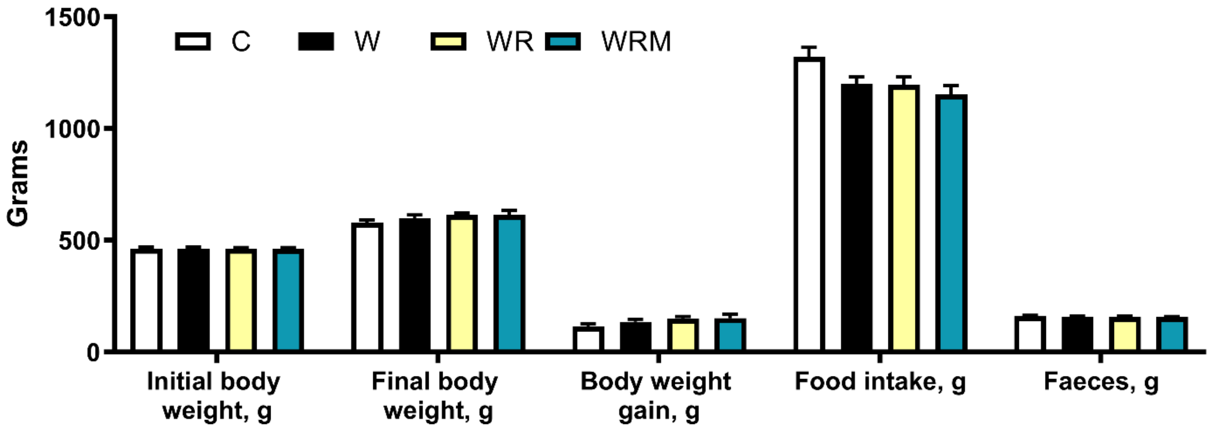

Figure S1. Metabolic monitoring during the 8 weeks of treatment in C, W, WR and WRM rats. Values are the means ± SEM of 8 different rats.
